# Intravitreal Use of a Bone Marrow Mononuclear Fraction (BMMF) Containing CD34+ Cells in Patients with Stargardt Type Macular Dystrophy

**DOI:** 10.1155/2020/8828256

**Published:** 2020-12-10

**Authors:** Carina Costa Cotrim, André M. Vieira Messias, Rodrigo Jorge, Rubens Camargo Siqueira

**Affiliations:** Department of Ophthalmology, Otorhinolaryngology and Head and Neck Surgery, Ribeirão Preto Medical School, University of São Paulo, Brazil

## Abstract

To assess the therapeutic potential and the safety of intravitreous use of a bone marrow mononuclear fraction (BMMF) containing CD34+ cells in patients with Stargardt type macular dystrophy. The study was conducted on 10 patients with Stargardt dystrophy with worse eye visual acuity ≤ 20/125. A bone marrow aspirate was obtained from all patients, and after processing in the cell therapy center (CTC), 0.1 ml of the intravitreous BMMF suspension was injected into the eye with worse visual acuity. A sham injection was performed in the contralateral eye. The patients were evaluated at baseline and one, three, and six months after the injection. All of them were submitted to measurement of best corrected visual acuity (BCVA), microperimetry, multifocal electroretinography (mfERG) and full field electroretinography (ffERG), autofluorescence (AF), and optical coherence tomography (OCT). Fluorescein angiography was also performed before and six months after the injection. All patients completed the six-month period of evaluation. Mean visual acuity of the treated eye was 1.1 logMAR (20/250) before intravitreous (IV) injection, 0.96 logMAR (20/200+2) one month after injection, and 0.92 logMAR (20/160-1) 3 months after injection. In the untreated eye, mean VA was 1.0 logMAR (20/200) at baseline and 0.96 logMAR (20/200+2) and 0.94 logMAR (20/160-2) one and three months after injection, respectively. In the treated group, VA at baseline ranged from best acuity of 20/125-1 to worst acuity of 20/640+2, going through 20/100+2 and 20/400 during the first month. In the untreated group, BCVA ranged from 20/100+2 to 20/400 at baseline and from 20/100 to 20/400 after one month. The results for the treated group differed significantly at all follow-up times, whereas no significant difference was observed in the untreated group. Regarding the mean sensitivity of microperimetry, although there was improvement throughout all months, a significant difference occurred only during the first month. In the untreated eye, there was no significant difference in any analysis. Angiofluoresceinography did not reveal neovessel formation or tumor growth. The remaining exams were used in order to aid the diagnosis. The results indicate that the use of intravitreous BMMF in patients with Stargardt dystrophy is safe and is associated with a discrete improvement of BCVA and microperimetry in the treated eye compared to the untreated one.

## 1. Introduction

Hereditary retinal dystrophies (HRD) are characterized by progressive loss of photoreceptor and/or retinal pigment epithelium function, with a consequent early impairment of patient vision [1]. Among the HRD, Stargardt disease is the most common juvenile macular dystrophy and a frequent hereditary cause of central visual dysfunction in young patients, typically during the first and second decades of life [2, 3]. The prevalence of Stargardt disease is 1 : 8,000 to 1 : 10,000 [4].

Stargardt macular dystrophy was first described in 1909 by the German doctor Karl Stargardt [5]. Its main inheritance is autosomal recessive and is associated with mutation of the ATP Binding Cassettte Retina-specific (ABCA4) gene, although there is high genetic heterogeneity [6]. The clinical signs and symptoms are characterized by bilateral loss of central vision and dyschromatopsia, with mapping revealing macular atrophy, beaten bronze macular lesions, and white-yellowish flecks corresponding to accumulation of lipofuscin at the retinal pigment epithelium (RPE) level [7–10]. In general, visual acuity (VA) may vary from 20/30 up to 20/200.

The diagnosis of Stargardt disease is based on clinical history, epidemiology, and complementary exams such as angiofluoresceinography, autofluorescence, (AF), optical coherence tomography (OCT), multifocal electroretinography (mfERG), and full field electroretinography (ffERG). The typical finding of angiofluoresceinography is “choroidal silence,” detected in 80% of cases and due to accumulation of lipofuscin in the RPE. Hyperfluorescent pisciform flecks are also observed [11, 12].

There is no treatment for Stargardt disease, but various therapeutic options are being tested, involving gene therapy and pharmacological and stem cell therapy [13]. There are different types of stem cells studied for degenerative diseases of the retina, including pluripontent cells such as embryonic stem cells (ESC), induced pluripotent stem cells (iPSCs), and multipotent cells, such as retinal progenitor cells (RPC) and bone marrow mononuclear fraction (BMMF) [14]. Schwartz et al. [15, 16] described the first clinical study of an RPE derived from ESC in patients in an advanced stage of Stargardt's disease and AMD. RPE cells were injected into the subretinal space in a pericentral region by pars plana vitrectomy (PPV). After four years of the study, Schwartz et al. [17] concluded that there was an improvement of visual acuity (VA) of up to 15 letters within six months.

Many investigators have assessed the safety and efficacy of the use of bone marrow hematopoietic cells for retinal diseases. In contrast to embryonic cells, which are administered by subretinal injection, bone marrow-derived cells are injected intravitreously in a simple procedure already used in clinical routine for other medications. Several studies have shown that these cells can act by releasing trophic factors with the ability to rescue suffering retinal cells [18–20].

The objective of the present study was to evaluate the safety and therapeutic potential of intravitreal use of a bone marrow mononuclear fraction containing CD34+ (BMMF) in ten patients with Stargardt macular dystrophy. The analyses were based mainly on the best corrected visual acuity (BCVA), the response of the microperimetry, and on angiofluoresceinography through the analysis of neovascularizations or growth of ocular tumors.

## 2. Materials and Methods

A prospective, nonrandomized, open study of changes in VA, microperimetry, OCT, and angiofluoresceinography induced by an intravitreous (IV) injection of BMMF containing CD34+ cells was conducted on ten patients with Stargardt macular dystrophy.

The protocol was approved by local and national review Ethical Committees, CONEP (Registration no. 15978 and amendment under registration CAAE 66839617.2.0000.5629), and followed the principles of the Statement of Human and Animal Rights, with registration no. NCT01518127 in Clinical Trials. Written informed consent was obtained from the patient(s) for their information to be published anonymously in this article. The patients were evaluated at the Retina and Vitreous outpatient clinic of the University Hospital, Ribeirão Preto Medical School, University of São Paulo (HCFMRP-USP) between January 2014 and December 2015. During selection, the patients were submitted to mfERG and ffERG, infrared (IR), and autofluorescence (FAF) for confirmation of the diagnosis. At baseline and one, three, and six months after IV injection, the patients were submitted to ophthalmological evaluation with BCVA, slit lamp biomicroscopy, tonometry, and retinal mapping with an indirect ophthalmoscope, in addition to OCT, microperimetry, IR, FAF, and angiofluoresceinography. Puncture of the posterior iliac crest and bone marrow aspiration were performed by the hematology team of HCFMRP-USP in a single procedure. The material was processed in the Cell Therapy Sector of the Blood Center of Ribeirão Preto. On the same collection day, IV injection was performed in the eye with worse VA. For statistical analysis, comparisons between groups for continuous data were performed by analysis of variance (ANOVA). To assess the variables (AV; BCEA, AT of the microperimetry), the covariance analysis was applied.

### 2.1. Inclusion and Exclusion Criteria

The inclusion criteria were presence of Stargardt disease, age from 18 to 50 years, and VA of 20/125 or worse. Exclusion criteria were IV injection of corticosteroids or other antiangiogenic drugs during the six months preceding the initial evaluation; media opacity that might interfere significantly with VA, confirmed by clinical ophthalmological evaluation or eye fundus documentation; intraocular surgery in the last three months; posterior vitrectomy or retinopexy with scleral introflection at any time; acute ocular infection, treatment of a face, skull, or neck region with ionizing radiation; allergy to fluorescein; alcoholism and use of drugs; medical or psychological conditions that would prevent the patient from concluding the study or from giving informed consent; a significant uncontrolled disease which might exclude the patient from the study in the opinion of the investigator; impediment of limited legal capacity; a history of malignant tumors at any time; and participation in another clinical study during the last 30 days.

### 2.2. Ophthalmological Evaluations

BCVA was measured according to the standardization recommended by the Early Treatment Diabetic Retinopathy Study Research Group [21], always by the same examiner during each visit. The patients were submitted to full ophthalmological examination including slit lamp biomicroscopy with and without dilatation, applanation tonometry, and indirect ophthalmoscopy with a 20D lens.

### 2.3. Multifocal and Full Field Electroretinography

The electroretinography exam was performed at the beginning of the study to help confirm the diagnosis of Stargardt.

### 2.4. Angiofluoresceinography, Infrared, and Autofluorescence

These exams were performed in all patients using the Spectralis equipment (Heidelberg Engineering, Heidelberg, Baden-Wurttemberg, Germany) at baseline, one and at three and six months after treatment. Angiofluoresceinography was performed in order to assess safety by the analysis of tumor growth and anomalous neovascularization.

### 2.5. Optical Coherence Tomography (OCT)

The patients were submitted to OCT at baseline and on the occasion of all subsequent visits using the Spectralis OCT system (Heidelberg Engineering, Heidelberg, Baden-wurttemberg, GER). The protocol used contained 10 horizontal sections (236 *μ*m between sections) centered on the fovea, for a total area of 20 × 15 degrees of the visual field (5.7 × 4.3 mm), with 25 frames for the means in each section.

### 2.6. Microperimetry

The exam was performed using the MAIA Centervue system (Centervue, Padova, ITA) at baseline and on the occasion of all visits. The images were obtained with a scanner laser (Scanner Laser Ophthalmoscopy). Sensitivity was measured from zero to 36 color-coded decibels (dB). The infrared image field was 36 × 36, and perimetry was performed in a 30 × 30 degree field with a luminance of 4 apostilb (asb). The test used was the Full-Threshold 4-2 test which assesses the retina in detail.

### 2.7. Material Collection

The technique used for bone marrow aspiration was similar to that described for oncologic and hematologic treatments for bone marrow transplantation. The procedure was carried out by a hematology team in the Sector of Bone Marrow Transplantation of HCFMRP-USP. After antisepsis with iodopovidone and anesthesia with lidocaine, the needle was introduced into the posterior iliac crest, and 5 to 10 ml bone marrow was aspirated from each patient with a heparinized syringe.

### 2.8. Cell Processing

**Figure 1:**
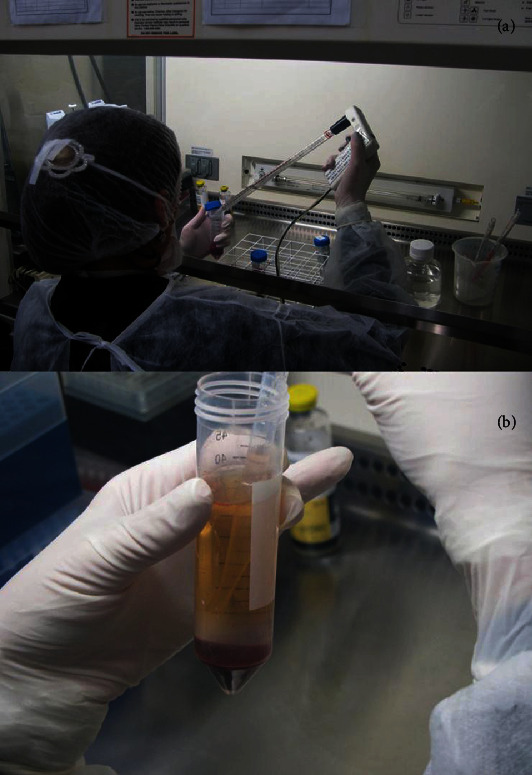
(a, b) Sequence of images showing the separation of mononuclear cells after centrifugation by Ficoll Hystopaque gradient.

The collected material was centrifuged on a Ficoll-Hypaque gradient (Amersham Pharmacia, a product licensed for human use) for the isolation of mononuclear cells. The mononuclear cell fraction obtained was resuspended in sterile saline solution and centrifuged again (Figure 1). The bone marrow mononuclear cells to be injected were characterized immonophenotypically by flow cytometry with a panel of monoclonal antibodies in order to determine the presence and the percentages of stem cells (CD34+) and of mature cells of hematopoietic and lymphoid lineages. The quantity of cells to be injected was, on average, 1.68 × 10^4^ cells in 0.1 ml.

All patients received a single IV injection of 0.1 ml BMMF containing CD34+ cells in the eye of worse VA. The technique followed established rules with periocular asepsis with topical PVPI and ocular asepsis with topical ophthalmic PVPI and the placement of a sterile surgical field and blepharostate. IV injection was performed with a 30 gauge needle in the upper temporal quadrant via pars plana (VPP) at 4 and 3.5 mm from the limb in phacic and pseudophacic eyes, respectively. Sham injection was applied to the contralateral eye for control by means of a disk pressed on the conjunctiva [22]. Antibiotic eyedrops (fourth generation quinolone) were prescribed for both eyes, with application four times a day for seven days.

## 3. Results

Ten patients with Stargardt muscular dystrophy were included in the study. All of them concluded six months of evaluation. The JMP® software, version 10.0.0., was used for all analyses (Table 1).

### 3.1. Visual Acuity

**Figure 2:**
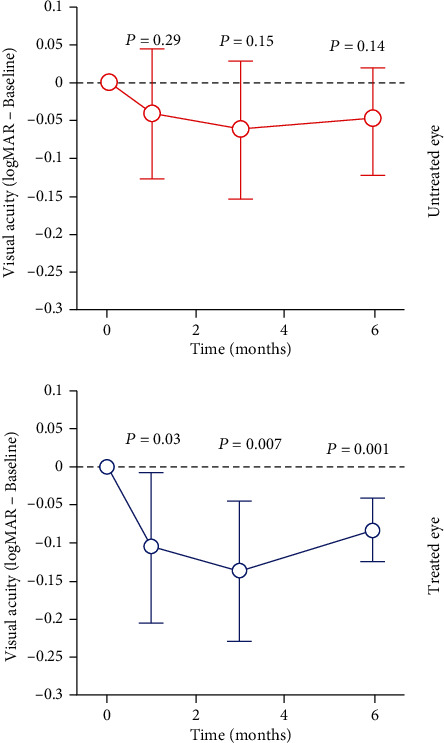
Graphical representation of the evolution of visual acuity in logMAR in relation to the follow-up time in months and the significance levels (*P*) of the treated eye × untreated eye.

Mean VA of the treated eye was 1.1 logMAR (20/250) before IV injection and 0.96 logMAR (20/200+2), 0.92 logMAR (20/160-1), and 0.98 logMAR (20/200+2) at one, three, and six months after the injection, respectively. In the eye with sham injection, mean VA was 1.0 logMAR (20/200) at baseline and 0.96 logMAR (20/200+2), 0.94 logMAR (20/160-2), and 0.96 logMAR (20/200+2) at one, three, and six months of follow-up, respectively (Table 2). A statistically significant difference was observed in the treated eye at all follow-up times, whereas no significant difference was observed in the untreated eye (Figure 2).

### 3.2. Angiofluoresceinography

No changes such as neovascularizations or tumor growth were observed throughout follow-up, thus demonstrating the achievement of the main objective of the present study, i.e., the safety of IV use of BMMF.

### 3.3. Electroretinography, Infrared, Autofluorescence, and OCT

All of these exams were of fundamental importance as diagnostic aids. The data obtained with them were not analyzed statistically.

### 3.4. Optical Coherence Tomography (OCT)

OCT did not reveal any changes in retinal anatomy.

### 3.5. Microperimetry

**Figure 3:**
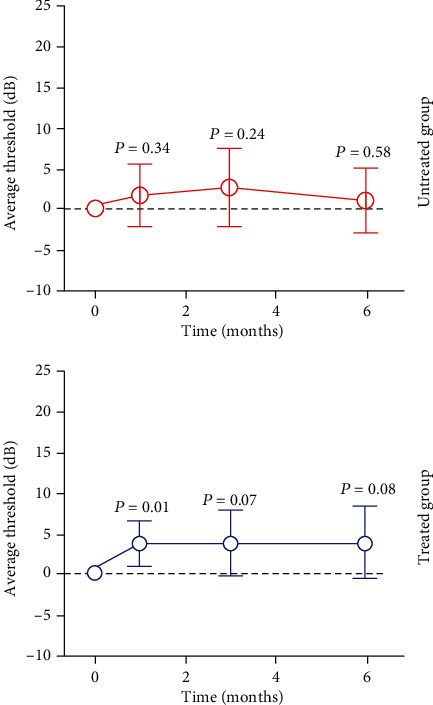
Graphical representation of the evolution of the mean sensitivity threshold in microperimetry in relation to the follow-up time in months and the different levels of significance (*P*) of the treated and untreated groups.

Two parameters were assessed in microperimetry: average sensitivity threshold and stability of fixation (bivariate contour ellipse area (BCEA)). Although average sensitivity improved throughout follow-up, a significant difference occurred only during the first month in the treated group. No significant difference was detected in the untreated eye at any time point (Figure 3).

**Figure 4:**
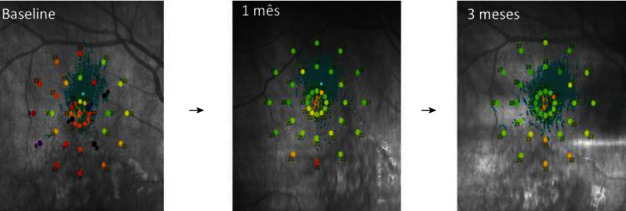
Microperimetry of patient #4 at baseline, 1 and 3 months after IV. Detail of the 36 points tested and their evolution.

The sensitivity threshold improved in all patients except patients #1 and #3, who showed a discrete reduction of sensitivity at three months and improvement in the sixth month. The patient #4 presented, in baseline microperimetry, 15 decibels and at 1 and 3 months, 26.2 and 26.6, respectively (Figure 4). The mean threshold sensitivity of the treated group was 15.0 decibels at baseline and 18.93 decibels at six months, while the mean threshold sensitivity of the untreated group was 16.2 decibels at baseline and 17.2 decibels at six months. Thus, improvement occurred in both treated and control eyes over the six-month period, although the initial threshold was higher and progressed with a discrete and lower improvement in the control than in the treated group. BCEA improved significantly in the treated group by the sixth month, whereas no difference was observed in the untreated group.

## 4. Discussion

BMMF is rich in cells whose main action is a trophic effect. Trophic or paracrine therapy is intended to improve the hostile retinal microenvironment in degeneration by releasing trophic factors and cytokines, increasing angiogenesis, reducing inflammation, and having an antiapoptotic effect, with remodeling of the extracellular matrix and activation of neighboring stem cells [23]. Hematopoietic cells are already being extensively used to reconstitute hematopoietic tissue in hematological diseases such as leukemia. Their benefits are currently being studied in different areas such as cardiology for the treatment of myocardial revascularization after ischemia, rheumatologic diseases such as Crohn disease and, more recently, in a multicenter study of patients with multiple sclerosis [23–25].

The main causes of intractable loss of vision are conditions associated with retinal dysfunction or degeneration [26]. These conditions are usually bilateral and affect the quality of life of the patients, as well as their productive capacity [27]. The objective of the present study was to investigate a probable currently unavailable therapy for patients with Stargardt macular dystrophy, the most common type of juvenile macular dystrophy [2].

Stem cells, characterized by unlimited capacity for proliferation and for the formation of new cells, as well as having a neuroprotective, immunomodulating, and antioxidant action, represent a promising perspective for the treatment of retinal dystrophies [28]. For the study of these cells, the eye has important advantages compared to any other organ since; in addition to being of easy access, it is divided into compartments, is immunologically protected, and requires a small cell volume for therapy [29].

The present study was conducted on ten patients aged 18 to 50 years with a previous diagnosis of Stargardt macular dystrophy and with VA of 20/125 or worse. The diagnosis was confirmed by clinical evaluation, ERG, autofluorescence, and infrared. All patients received an IV injection containing 0.1 ml BMMF in to the eye with the worse VA and a sham injection into the contralateral eye. No complications such as infections, retinal detachment, uveitis, or tumor formation were observed in any of the patients, with confirmation of safety of the application, which was the main objective of the present study. Previous studies have also confirmed this safety for other retinal diseases. Jonas et al. [30] applied an IV injection of bone marrow-derived mononuclear cells to three patients with respective diagnoses of age-related macular degeneration (AMD), diabetic retinopathy, and glaucoma, with no complications. Siqueira [19] reported the same safety for three patients with retinitis pigmentosa and two patients with cone-rod dystrophy. In a phase I study of six patients (six eyes) with ischemia or retinal degeneration. Park et al. [31] did not detect any side effects. Cotrim et al. [32] assessed the safety of the IV use of the same cells in ten patients with dry ARMD. These are the major world studies of the use of bone marrow-derived adult stem cells.

A statistically significant difference in BCVA was observed in the treated group at all time points, whereas no significant difference was observed in the untreated group. Four of the ten patients gained one vision line at three months, with the greatest gain being 4 lines and the smallest one letter. It is important to point out that there is uncertainty about the time these cells remain in the intraocular position. In a preclinical study on mice, Otaniet al [33]. demonstrated the permanence of these cells for as long as six months. Using immunohistochemical analysis, Park et al. [34] demonstrated the presence of CD34+ cells incorporated into the retinal vasculature of mice with acute ischemia induced one day after IV injection and their permanence up to six months. In a later clinical study, Park et al. [31] detected by adaptive optic OCT the presence of hyperreflexive cells that may have corresponded to CD34+ cells one month after IV and their permanence in the retina up to the last assessment at six months, although in smaller amounts. Caballero et al. [35] showed a rapid incorporation of CD34+ cells in a murine model of induced retinal ischemia. Thus, the functional evaluations of highest value in the present study were those up to six months but mainly up to the third month, when these cells are believed to reach the peak of their function and due to the fact that the cited studies have emphasized their permanence up to the sixth month. Siqueira et al. [36] observed improved vision in their five patients, although their inclusion criterion was vision worse than 20/200 and more advanced disease. Park et al. [31] also observed VA improvement of 3 to 65 letters in their six patients with diagnoses such as Stargardt disease, ARMD, occlusion of the central artery and vein of the retina, and retinitis pigmentosa. Cotrim et al. [32] detected improved VA ranging from three to 19 letters in patients with dry ARMD.

During the first three months, eight patients showed improvement of the sensitivity threshold, but a clinically significant difference was observed only during the first month. No significant difference was observed in the untreated eye at any time point. Park et al. [31] compared the mean sensitivity threshold (decibels) of microperimetry in six patients submitted to IV injection, with improved sensitivity in three of them; one remained stable, and two showed a perceptible decline after six months of follow-up. Cotrim et al. [32] detected improvement of the sensitivity threshold of patients with ARMD at all monthly time points, with a significant result being observed only after the sixth month, a fact leading to the question of the learning effect. Regarding BCEA, there was a significant improvement of fixation by the sixth month, with no difference occurring in the control eye. Cotrim et al. [32] reported improvement of BCEA throughout their study, although without statistical significance.

Angiofluoresceinography was an important exam for the assessment of the safety of BMMF use. The procedure was applied to all patients and did not reveal the formation of choroidal or retinal neovascularization or of tumors. Although some studies have demonstrated improved retinal perfusion with hematopoietic stem cells, these vascular changes were not observed in the present study [33].

Regarding OCT, no changes in retinal anatomy or thickness were observed. Atrophy of retinal layers was expected in the disease and was maintained throughout follow-up. A possible hypothesis would be that the spectral domain may not yet be sufficient to reveal anatomical changes at the cellular level. Some patients with retinitis pigmentosa may develop macular edema, with OCT being of fundamental importance for this analysis. Siqueira et al. [36] evaluated a case of macular edema resolution in the eye injected IV with BMMF compared to the control eye. The patient had already been treated with acetazolamide for a long period of time, with no results. The use of new technologies such as adaptive optics may contribute to a better analysis at the cellular level.

## 5. Conclusions

On the basis of the results obtained, we conclude that autologous BMMF containing CD34+ cells proved to be safe for IV application to patients with Stargardt macular dystrophy during the study period, supporting the results of previous studies using the same cells for other ocular diseases. The study involved a small number of patients, with its major objective being the assessment of the safety of the use of these cells. It is important to increase the number of patients in order to reinforce the improvement data obtained. Some questions are still unanswered, such as how long the cell survives in the intraocular medium, when the trophic effect starts, and whether or not freezing the material for future applications to the same patient would jeopardize the effect of the cells. Taking into account the main trophic effect of BMMF, a more significant improvement and ocular protection might have been observed in earlier phases of the disease when most retinal cells are still suffering but are not dead. Thus, in addition to studying a larger number of patients, it would be important to include those with better acuity. Another important factor would be the inclusion of technology such as adaptive optics in order to obtain more details about the adhesion of these cells to the retina, their behavior, and their time of permanence.

## Figures and Tables

**Table 1:** Patient data such as gender, treated eye, age, age of diagnosis, and duration of the disease.

Patients	Gender	Treated eye	Age	Age of diagnosis	Duration of the disease
#1	F	LE	48	30	18
#2	F	RE	23	13	10
#3	F	RE	27	17	10
#4	F	LE	26	21	5
#5	F	RE	33	25	8
#6	M	LE	40	25	15
#7	M	LE	45	25	20
#8	M	LE	33	22	11
#9	M	RE	28	22	6
#10	F	RE	27	17	10

**Table 2:** Visual acuity measured before IV injection, three and six months after. Description of the number of letters won per patient (patient #1 to #10).

Patients	Visual acuity baseline	Letters	Visual acuity 3 months	Letters	Visual acuity 6 months
#1	20/160-2	4	20/160+2	2	20/160
#2	20/400	14	20/200-1	7	20/320+2
#3	20/400	7	20/320+2	0	20/400
#4	20/125-2	1	20/125-1	1	20/125-1
#5	20/125-2	4	20/125+2	7	20/100
#6	20/160-1	3	20/160+2	6	20/125
#7	20/640+2	22	20/200-1	7	20/400
#8	20/160-2	4	20/160+2	4	20/160+2
#9	20/125-1	4	20/100-2	4	20/100-2
#10	20/400-1	6	20/320	6	20/320

## Data Availability

All data from these studies are only available on the website of the University of Sao Paulo (https://www.teses.usp.br/).
